# Gene Expression in Uninvolved Oral Mucosa of OSCC Patients Facilitates Identification of Markers Predictive of OSCC Outcomes

**DOI:** 10.1371/journal.pone.0046575

**Published:** 2012-09-28

**Authors:** Pawadee Lohavanichbutr, John Houck, David R. Doody, Pei Wang, Eduardo Mendez, Neal Futran, Melissa P. Upton, F. Christopher Holsinger, Stephen M. Schwartz, Chu Chen

**Affiliations:** 1 Program in Epidemiology, Fred Hutchinson Cancer Research Center, Seattle, Washington, United States of America; 2 Program in Biostatistics and Biomathematics, Fred Hutchinson Cancer Research Center, Seattle, Washington, United States of America; 3 Department of Otolaryngology – Head and Neck Surgery, University of Washington, Seattle, Washington, United States of America; 4 Surgery and Perioperative Care Service, VA Puget Sound Health Care System, Seattle, Washington, United States of America; 5 Clinical Research Division, Fred Hutchinson Cancer Research Center, Seattle, Washington, United States of America; 6 Department of Pathology, University of Washington, Seattle, Washington, United States of America; 7 Department of Otolaryngology – Head and Neck Surgery, MD Anderson Cancer Center, Houston, Texas, United States of America; 8 Department of Epidemiology, University of Washington, Seattle, Washington, United States of America; The University of Texas MD Anderson Cancer Center, United States of America

## Abstract

Oral and oropharyngeal squamous cell carcinomas (OSCC) are among the most common cancers worldwide, with approximately 60% 5-yr survival rate. To identify potential markers for disease progression, we used Affymetrix U133 plus 2.0 arrays to examine the gene expression profiles of 167 primary tumor samples from OSCC patients, 58 uninvolved oral mucosae from OSCC patients and 45 normal oral mucosae from patients without oral cancer, all enrolled at one of the three University of Washington-affiliated medical centers between 2003 to 2008. We found 2,596 probe sets differentially expressed between 167 tumor samples and 45 normal samples. Among 2,596 probe sets, 71 were significantly and consistently up- or down-regulated in the comparison between normal samples and uninvolved oral samples and between uninvolved oral samples and tumor samples. Cox regression analyses showed that 20 of the 71 probe sets were significantly associated with progression-free survival. The risk score for each patient was calculated from coefficients of a Cox model incorporating these 20 probe sets. The hazard ratio (HR) associated with each unit change in the risk score adjusting for age, gender, tumor stage, and high-risk HPV status was 2.7 (95% CI: 2.0–3.8, p = 8.8E-10). The risk scores in an independent dataset of 74 OSCC patients from the MD Anderson Cancer Center was also significantly associated with progression-free survival independent of age, gender, and tumor stage (HR 1.6, 95% CI: 1.1–2.2, p = 0.008). Gene Set Enrichment Analysis showed that the most prominent biological pathway represented by the 71 probe sets was the Integrin cell surface interactions pathway. In conclusion, we identified 71 probe sets in which dysregulation occurred in both uninvolved oral mucosal and cancer samples. Dysregulation of 20 of the 71 probe sets was associated with progression-free survival and was validated in an independent dataset.

## Introduction

Oral and oropharyngeal squamous cell carcinomas (OSCC) are among the most common cancers, with approximately 400,000 new cases and 200,000 deaths worldwide in 2008 (http://www-dep.iarc.fr/). Approximately 40,000 new cases and almost 8,000 deaths from OSCC are estimated to occur in the United States in 2012 [1]. The overall 5-yr survival rate of OSCC patients is approximately 60% [1]. The prognosis of OSCC patients is adversely influenced by the development of recurrent cancer, which occurs in 5–50% of patients [2]–[4]. Better prediction of which patients are most at risk for recurrence or disease progression is needed. Several factors have been found to be predictive of the development of recurrent OSCC, including tumor stage, tumor depth, nodal status, lymphovascular or perineural invasion, positive surgical margins, and extracapsular spread [5]–[8]. However, further improvement in the prediction of risk for recurrence or disease progression could help physicians identify patients who need more aggressive treatment or more frequent follow-up. Genes that play roles in the progression of normal tissue to cancer may serve as markers to predict recurrence or disease progression of OSCC patients.

Based on the field cancerization concept proposed by Slaughter et al in 1953 [9], the changes in the mucosa of the entire upper aerodigestive tract may be the result of long term exposure to carcinogens and may explain the occurrence of local recurrence or second primary disease. The field cancerization concept was supported by subsequent studies which found abnormal histologic and molecular features in the uninvolved, clinically normal, oral mucosae of OSCC patients [10]–[18]. A number of studies have shown alterations at a molecular level, such as loss of heterozygosity (LOH) at 3p, 9p, and 17p [10], gain of chromosome region 20q13.33, 7p22.2-pter, 11p15.5-pter, and 16p13.3-pter [11], and p53 mutation [10], [12] in the uninvolved oral mucosae, either adjacent to or distant from the tumor of OSCC patients. There is also evidence of increased expression of some genes such as epidermal growth factor receptors [13], cyclin D1 and mindbomb E3 ubiquitin protein ligase 1 [14], and cytokeratins [15] in the uninvolved oral mucosae of OSCC patients.

In addition to the studies of molecular changes in the uninvolved oral mucosae of OSCC patients, there have been hundreds of studies reporting on the molecular changes in the oral cancer tissues, either at an individual gene level or a genome-wide level. For example, there have been reports of LOH on Chromosome 1p31, 3p25-p26, 4q25, 5q21-22, 8p21-23, 9p21-22, 10 at D10S202 and DD10S217, 11q, 14q, 17p, 20q12-13.1, and 21q11.1 in OSCC samples [19]–[31]. Studies using array comparative genomic hybridization (CGH) further expand the knowledge of gains and losses of chromosomal regions across the genome. Gains at chromosomal regions 1q23, 3q23, 3q26, 5p15.2, 5p15.33, 7p11, 7p12.3-13, 7p22.3, 7q21.2, 7q35, 8q21.1-24.3, 8q24, 9q34.3, 11q13, 14q23,16p13.3, 19q12, 19q13, 20q13, and losses at 2p15, 3p21-3p12, 3p22, 3p14, 4q34.3, 4q35.2, 8p32,10p12, 16q23.2, 18q21-q23 in OSCC samples have been detected using array CGH [32]–[34]. Several researchers have used proteomics to identify diagnostic [35]–[41] or prognostic [42]–[44] biomarkers for OSCC; however, these studies were either small or had no external validation.

With the advent of a high-throughput microarray technology, investigation of gene expression on a genome-wide level has become feasible and routine. Microarray studies usually result in a list of many genes; further definition of the functions of or pathways involving these genes could provide additional knowledge about OSCC. A gene expression profile, if validated in multiple, well-designed, independent studies, could also be developed as a useful clinical test as demonstrated for breast cancer [45]. Prior studies, including ours, have reported many genes differentially expressed between OSCC and normal tissue [46]–[51]. However, these studies compared OSCC to either uninvolved oral mucosae of OSCC patients or normal oral mucosae from people without cancer. To the best of our knowledge, no study has compared genome-wide gene expression profiles of normal oral mucosae from non-cancerous patients, uninvolved oral mucosae from OSCC patients, and OSCC samples in the same study. As proposed by Braakhuis [52], oral carcinogenesis can be viewed as a multistep process; from normal tissue to a patch, which progresses to a field, and finally to an invasive carcinoma with additional genetic alterations in each step. Identifying the changes in gene expression in these steps may help advance our understanding of the disease progression process and lead to discovery of markers to predict disease progression. The purpose of the current study is to identify genes that are dysregulated in uninvolved oral mucosae from OSCC patients compared with normal oral mucosae from patients without cancer, and show further dysregulation in cancer tissue. We believe that these genes may play an important role in the progression of OSCC, and we tested our hypothesis by determining whether the expressions of dysregulated genes are associated with disease progression or OSCC-specific mortality.

## Methods

### Ethics Statement

This study was conducted with written informed consent of the study participants and the approval of the Institutional Review Boards of the Fred Hutchinson Cancer Research Center, the Veterans Affairs Puget Sound Health Care System, and the MD Anderson Cancer Center.

### Study Population

Eligible cases are patients with first primary OSCC treated at one of the three University of Washington-affiliated medical centers in Seattle, WA from December 2003 to March 2010. Eligible controls are patients without OSCC who had oral surgery, such as tonsillectomy or uvulopalatopharyngoplasty, at the same institutions and during the same time period in which the OSCC cases were treated.

### Data and Tissue Collection

Each patient was interviewed using a structured questionnaire regarding his or her demographic, medical, and lifestyle history, including tobacco and alcohol use. Data on tumor characteristics were obtained from medical records. The data on tumor recurrence were obtained from telephone interview and confirmed by medical record abstraction if patients reported having a tumor recurrence. If patients were not followed at one of the three University of Washington-affiliated medical centers, we attempted to obtain medical record from their physicians. Vital status was obtained from Social Security Death Index (SSDI) and Fred Hutchinson Cancer Research Center's Cancer Surveillance System (CSS), which is part of the Surveillance, Epidemiology, and End Results (SEER) program of the National Cancer Institute. Death certificates and medical records, if available, were reviewed by otolaryngologists to determine the cause of death. The last search for vital status of all patients was in September 2011.

The tumor samples and uninvolved oral mucosa were obtained from OSCC patients at the time of resection prior to chemo/radiation therapy, if any. The uninvolved oral mucosa was collected either from the opposite side of the tumor or from the same side but far from the tumor margin. From controls we obtained normal mucosa from buccal, uvula or anterior tonsillar pillar, the latter with effort to avoid surrounding lymphoid tissues. Between December 2003 and September 2008, the tissue samples were soaked in RNAlater™ immediately after surgical removal and transferred to long term storage at −80°C prior to use. After September 2008, the tissue samples were flash frozen in liquid nitrogen immediately after surgical removal.

From December 2003 to March 2010, we recruited 291 cases and 58 controls. Gene expression data from tumor samples of 167 cases and normal oral mucosae of 45 controls were generated in our previous study [46] using samples treated with RNAlater™. For comparability, in the current study we only used uninvolved oral mucosa samples that had been treated with RNAlater™. As we had limited funds to measure the gene expression in uninvolved oral mucosa, we could only examine a subset of these samples. We suspected that the gene expression of uninvolved oral samples from patients with and without local recurrence may be different. Uninvolved oral mucosa of patients who later developed local recurrence may be more likely to contain genes that are associated with disease progression, and thus oversampling of uninvolved oral mucosa from patients with local recurrence might enhance the opportunity to detect genes that are associated with disease progression. We therefore included uninvolved oral samples from all patients who had recurrent/second primary OSCC as of March 2010 (n = 29). We then used stratified sampling to select another 29 OSCC patients who had not had a recurrent/second primary OSCC, but otherwise had a similar follow-up time distribution as the 29 patients with recurrent/second primary OSCC. Forty-nine of the 58 selected OSCC patients also provided tumor samples that had already been processed in the previous study (part of the 167 tumor samples).

### Laboratory Methods

The DNA and RNA from each specimen were simultaneously extracted using the TRIzol method (Invitrogen, Carlsbad, CA). RNA was further purified using RNeasy mini kit (Qiagen, Valencia, California) and then converted to double-stranded complementary DNA (cDNA) using a GeneChip Expression 3′-Amplification One-cycle DNA Synthesis Kit (Affymetrix). The cRNA was produced from cDNA and was hybridized to a U133 2.0 Plus GeneChip (Affymetrix) as previously described [46]. HPV DNA was tested using a nested PCR based protocol and confirmed by LINEAR ARRAY HPV Genotyping Test (Roche, Indianapolis, IN) under a research use only agreement as described in Lohavanichbutr et al [53].

### Quality Control

For quality control, we re-extracted and processed two tumor samples, whose genome-wide gene expression had been assessed in our previous study, along with the uninvolved oral samples. We used Pearson correlation to determine whether the previous and new gene expression were comparable. We found a good correlation between samples previously processed and samples processed along with uninvolved oral samples. The Pearson's correlation coefficients for all probe sets of the two pair were 0.96 and 0.97.

The quality of the hybridized arrays was evaluated using the “affyQCReport” and “affyPLM” software in the Bioconductor package (http://bioconductor.org/). This included evaluation of RNA degradation and detection for possible outlier array. We examined 58 arrays of uninvolved oral samples separately and also together with 212 arrays (167 from tumor samples and 45 from normal oral samples) previously processed in order to detect a batch effect. All 58 arrays for uninvolved oral samples passed quality control and no batch effect was observed.

### Statistical Analyses

#### Assessment of Differential Gene Expression

**Figure 1:**
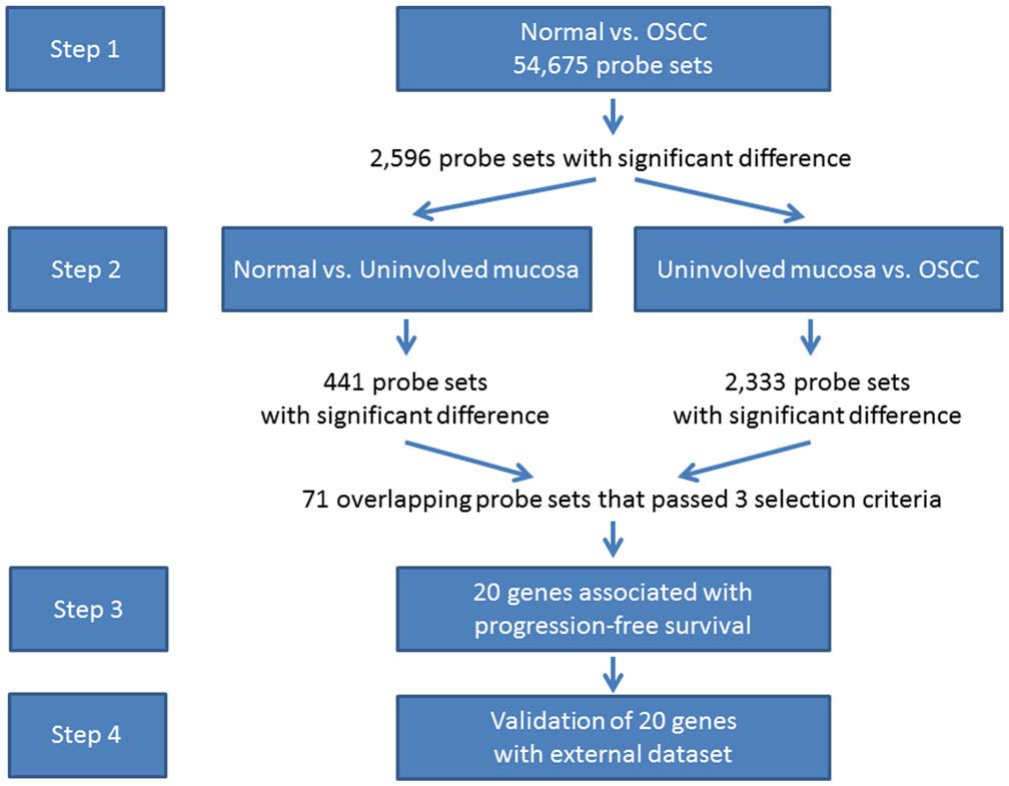
Steps for statistical analyses. Step 1, compared normal mucosae from non-cancerous patients to cancer tissues from OSCC patients to identify genes associated with OSCC and to reduce the number of genes for further comparison. Step 2 had two comparisons: 1) compared normal mucosae from non-cancerous patients to uninvolved mucosae from OSCC patients, 2) compared uninvolved mucosae from OSCC patients to cancer tissues. We then selected the genes that overlapped between the two comparisons and passed three selection criteria (i. the Bonferroni adjusted p-value must be less than 0.05 in both comparisons; ii. the magnitude of the difference in expression level must be greater than one standard deviation of the expression in the uninvolved oral samples; iii. the direction of the coefficients of each gene must be the same in both comparisons, i.e. the coefficients must be positive in both comparisons for up-regulated genes, and must be negative for both comparisons for down-regulated genes. Step 3 among the genes selected from step 2, we identified those that were associated with progression-free survival. In Step 4, we validated the genes identified in Step 3 using an independent external dataset.

All 270 CEL files were normalized using the RMA algorithm in Partek® Genomics Suite™ software. Figure 1 shows steps of statistical analyses. We first identified “OSCC-related genes” by comparing gene expression profiles of normal oral samples from 45 controls to tumor samples from 167 OSCC cases using ANOVA implemented in Partek® Genomics Suite™ software, adjusting for age (continuous variable), sex (male vs. female), cigarette-smoking (current smoker vs. never/former smoker), alcohol use (current vs. never/former alcohol use) and HPV status (high risk vs. negative/low risk). We set the false discovery rate at 0.05 and required at least a 2-fold difference in gene expression as criteria for differential expression. The purpose of this first step is to reduce the number of genes for further comparison. The next step is to identify genes, among the “OSCC-related genes”, that show dysregulation in a field of carcinogenic exposure (uninvolved oral mucosa) and increased level of dysregulation in cancer stage. To identify these genes, we used linear regression to compare the gene expression level between 45 normal oral samples and 58 uninvolved oral samples, and to compare the gene expression level of 58 uninvolved oral samples to that of 167 tumor samples. We used three criteria to select the gene list: 1) the Bonferroni adjusted p-value must be less than 0.05 in both comparisons; 2) the magnitude of the difference in expression level must be greater than one standard deviation of the expression in the uninvolved oral samples; 3) the direction of the coefficients of each gene must be the same in both comparisons, i.e. the coefficients must be positive in both comparisons for up-regulated genes, and must be negative for both comparisons for down-regulated genes. The analyses were performed using STATA 11.1 (StataCorp, College Station, TX).

#### Evaluation of Gene Expression Profile in relation to Disease Outcome

To determine whether the selected genes are associated with disease progression or death due to OSCC, we performed a Cox regression with robust standard error on each selected gene adjusting for age, gender, high-risk HPV status, and tumor stage (stage I/II vs. stage III/IV). In this study, disease progression is defined as a persistence or recurrence of squamous cell carcinoma in oral cavity, oropharynx, or in the head and neck area. Patients who were alive as of September 2011 or patients who died with other causes were censored at the time of last known disease status either at the last follow-up interview or at the last clinic visit. We used a Bonferroni adjusted p-value of 0.05 as a criterion to select genes that are associated with disease progression/OSCC-related death. We then built a Cox regression model with the genes associated with disease progression/OSCC-related death and used coefficients from this model to calculate a risk score for each patient.

#### Validation using External Dataset

An independent dataset of 74 frozen tumor samples from OSCC patients treated at the MD Anderson Cancer Center (MDACC) was used for validation. The 74 tumor samples were hybridized at the MDACC to the same type of Affymetrix array as used in our study. We normalized the CEL files using RMA algorithm in Partek® Genomics Suite™ software. A risk score for each patient was calculated using coefficients from a Cox regression model from our study. We then investigated the association between risk score and disease progression/OSCC-related death using a Cox regression analysis adjusting for age (continuous variable), gender (male vs. female), and tumor stage (I/II vs, III/IV). We compared the model with tumor stage alone and tumor stage plus risk score using a log likelihood ratio test. The patients were divided into three equal size groups based on the risk scores (low, medium, and high). We then used a Kaplan-Meier method to compare progression free survival for patients in each group.

#### Validation of gene expression using qRT-PCR

To affirm our findings based on the Affymetrix array, we conducted qRT-PCR to assess the gene expression of the top five genes (*OCC1*, *DSE*, *ACTN1*, *RRAS2*, and *ITGA3*). In brief, qRT-PCR was performed using 7.5 ng purified total RNA from each of 48 samples (a subset of 270 samples) using the QuantiTect SYBR Green RT-PCR kit (Qiagen, Valencia, CA) and bioinformatically validated QuantiTect primers (Qiagen, Valencia, CA). Each sample was run in triplicate on a 7900 HT Sequence Detection System (ABI, Foster City, CA). The cycling conditions were: 30 min, 50°C; 15 min, 95°C; 40 cycles of 15 sec at 94°C, 30 sec at 55°C, and 30 sec at 72°C. Beta-Actin (*ACTB*) was used as a reference gene for normalization. We used Pearson's correlation to determine the correlation between the Affymetrix gene expression values and the Ct (cycle threshold) values from qRT-PCR.

#### Pathway analyses

We used Gene Set Enrichment Analysis (GSEA) [54] to investigate pathways of the genes dysregulated in uninvolved oral samples and tumor samples. GSEA computes the overlap between genes of interest and the gene sets in the Molecular Signatures Database (MSigDB). The gene sets of the pathways in the MSigDB are derived from three pathway databases: the Biocarta pathway database (www.biocarta.com), the KEGG pathway database (www.genome.jp/keg), and the Reactome pathway database (www.reactome.org). The p-value indicating the significance of the overlap was calculated based on the hypergeometric distribution (identical to the corresponding one-tailed version of Fisher's exact test). We used 0.05 for a p-value cutoff. We also used GSEA to compare the pathways of the genes that we found in this study to the 131 genes that we previously reported to be associated with survival of OSCC patients [55].

## Results

Selected characteristics of the study participants are showed in Table 1. Compared to controls, OSCC patients were more likely to be older, white, and current smokers. Approximately two-thirds of the cases had an advanced stage tumor.

**Table 1:** Selected characteristics of OSCC patients by sample type and controls, University of Washington Affiliated Medical Centers, 2003–2010.

	OSCC				Control	
	Tumor sample		Uninvolved oral sample		Normal oral sample	
	(n = 167*)		(n = 58)		(n = 45)	
Characteristic	n	%	n	%	n	%
**Age**						
19–39	7	4.2	4	6.9	17	37.8
40–49	26	15.6	10	17.3	14	31.1
50–59	57	34.1	18	31.0	5	11.1
60–90	77	46.1	26	44.8	9	20.0
**Gender**						
Male	120	71.9	41	70.7	32	71.1
Female	47	28.1	17	29.3	13	28.9
**Race**						
White	152	91.0	54	93.1	31	68.9
Non-white	15	9.0	4	6.9	14	31.1
**Smoking status**						
Never/Former	86	51.5	37	63.8	33	73.3
Current	81	48.5	21	36.2	12	26.7
**Drinking status**						
Never/Former	55	33.5	23	39.7	11	25.0
Current	109	66.5	35	60.3	33	75.0
Unknown	3				1	
**AJCC staging**						
I	39	23.3	15	26.3		
II	16	9.6	9	15.8		
III	22	13.2	8	14.0		
IV	90	53.9	25	43.9		
Unknown			1			

* 49 of 167 OSCC patients provided both tumor tissue and uninvolved oral tissue.

### Gene Selection

In the ANOVA analysis to identify “OSCC-related genes”, we found 2,596 probe sets differentially expressed between 167 tumor samples and 45 normal oral samples from controls, using the criteria of a FDR of 0.05 and at least a two-fold difference in the expression level. The result of linear regression comparing gene expression level of 2,596 probe sets between 45 normal oral samples from controls and 58 uninvolved oral samples from OSCC cases, and between 58 uninvolved oral samples and 167 tumor samples (both from OSCC cases), showed that 60 probe sets were significantly and consistently up-regulated and 11 probe sets were significantly and consistently down-regulated in both comparisons, using the three criteria described in the Methods section. The list of the 71 probe sets is presented in Table S1.

### Survival analyses

We excluded nine of 167 cases who died within 30 days of surgery (due to complication of surgery) or who had been followed for less than 30 days. Among 158 patients included in the survival analyses, 70 had disease progression/OSCC-related death. The follow-up time for patients without progression/OSCC-related death ranged from 3.6 to 83.9 months, with a median follow-up time of 43.3 months. The result of Cox regression analyses of each of the 71 probe sets adjusting for age, sex, tumor stage, and high-risk HPV status showed 20 of 71 probe sets significantly associated with disease progression/OSCC-related death with a p-value<0.0007 (Table 2). We then built a prediction model based on the Cox regression model incorporating the 20 probe sets. A coefficient of each probe set (Table 2) was multiplied with the expression of that probe set and summed to be a risk score for each patient. The risk score ranged from 11.0 to 18.6 (mean 14.9, standard deviation 1.2). In our study, each unit increase in risk score was associated with a hazard ratio of 2.7 for disease progression/OSCC-related death after adjusting for age, gender, tumor stage, and high-risk HPV status (95% CI: 2.0–3.8, p-value<0.001).

**Table 2:** Gene expression of 20 probe sets associated with disease progression or death due to OSCC, University of Washington Affiliated Medical Centers, 2003–2010.

Probe ID	Gene Symbol	HR*	95% CI		p-value	Coefficient in the Cox model**
225105_at	OCC1	1.9	1.5	2.4	4.00E-07	0.46727
218854_at	SART2	3.0	1.9	4.6	5.80E-07	0.22577
208636_at	ACTN1	2.3	1.6	3.3	1.90E-06	0.47392
212589_at	RRAS2	2.5	1.7	3.8	6.80E-06	0.61303
201474_s_at	ITGA3	2.1	1.5	2.9	6.90E-06	−0.10248
201976_s_at	MYO10	1.9	1.4	2.6	7.70E-06	0.16939
204334_at	KLF7	2.1	1.5	2.9	2.30E-05	0.59466
214853_s_at	SHC1	2.7	1.7	4.3	6.00E-05	0.59933
1552277_a_at	MSANTD3	2.2	1.5	3.3	6.10E-05	0.63538
202896_s_at	SIRPA	2.3	1.5	3.5	6.10E-05	−0.48287
225795_at	C22orf32	0.3	0.2	0.6	7.70E-05	−0.60779
202872_at	ATP6V1C1	2.6	1.6	4.2	9.00E-05	0.44730
205122_at	TMEFF1	1.5	1.2	1.8	1.00E-04	−0.16921
213139_at	SNAI2	2.1	1.4	3.0	1.00E-04	−0.59571
228914_at	MSANTD3-TMEFF1	2.2	1.4	3.3	2.30E-04	−0.29491
235492_at	RNF217	1.9	1.3	2.7	2.80E-04	−0.23651
221898_at	PDPN	1.5	1.2	1.9	2.90E-04	−0.19016
202599_s_at	NRIP1	1.9	1.3	2.7	3.00E-04	0.16372
206581_at	BNC1	1.7	1.3	2.3	4.80E-04	−0.25078
1558152_at	LOC100131262	1.5	1.2	2.0	6.10E-04	−0.15511

* Hazard ratio of each gene from Cox regression analysis adjusting for age, sex, tumor stage, and high-risk HPV status.

** Cox model incorporating 20 probe sets, used for calculating a risk score.

### Analyses of 20 probe sets in an independent dataset

**Figure 2:**
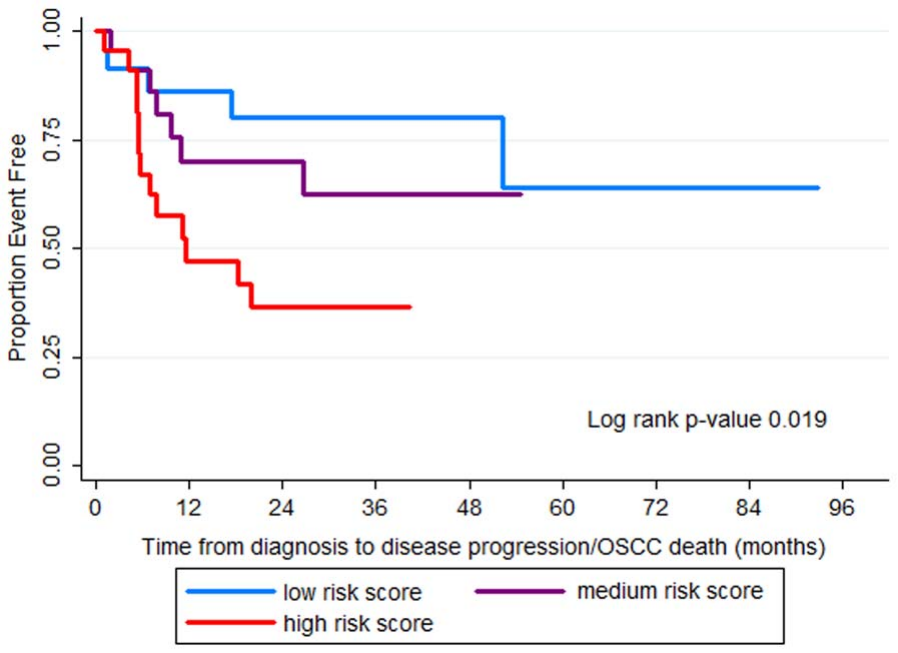
Kaplan-Meier survival curves. Kaplan-Meier curves comparing progression free survival of 69 OSCC patients in the MDACC dataset with low, medium, and high risk score.

Among 74 OSCC patients from the MDACC, five patients had follow-up time less than 30 days and were excluded from the survival analyses. The age range of the patients was 22 to 84 years, with an average age of 58.2 years. The majority of patients had stage III or IV disease (73.9%). Twenty-five of 69 patients had disease progression/OSCC-related death. The follow-up time for 69 patients ranged from one month to 92.7 months. The median follow-up time for patients without events was 22.7 months (range 1.6 to 92.7 months). A risk score for each patient was calculated using coefficients of the prediction model from the University of Washington data and the expression values from each MDACC patients as described above. The risk score ranged from 14.8 to 20.4 (mean 17.8, standard deviation 1.4). The crude hazard ratio (HR) for each unit increase in the risk score was 1.63 (95% CI: 1.16–2.29, p-value 0.004). The hazard ratio associated with a risk score after adjusting for age, gender, and tumor stage was 1.59 (95% CI: 1.13–2.23, p-value 0.008). The hazard ratio for each variable in the model is shown in Table 3. Data on HPV status were not available for the MDACC: therefore we could not adjust for HPV status. Higher tumor stage (stage III/IV) was associated with a higher risk of disease progression/OSCC-specific mortality; however, it did not reach statistical significance in the MDACC (crude HR 2.3, 95% CI: 0.79–6.75, p-value 0.13, HR adjusted for age, gender, and risk score 1.65, 95%CI: 0.53–5.19, p-value 0.39). The prediction model incorporating the risk score and tumor stage provided a better fit to the data than the model with tumor stage alone (log likelihood ratio test p-value = 0.006); however, it was not better than the model with risk score alone (log likelihood ratio test p-value = 0.3). The HR adjusted for age, gender, and tumor stage for patients with medium risk score and high risk score compared to patients with low risk score was 1.79 (95% CI: 0.54–5.91, p-value 0.34) and 3.67 (95% CI:1.2–11.2, p-value 0.02), respectively. Kaplan-Meier curves provided additional illustration of the progression-free survival of patients in each group (Figure 2). Patients with high risk score had poorer progression free survival than patients with medium and low risk score, with a Log-rank p-value of 0.019.

**Table 3:** Association of a risk score calculated from a 20 probe sets prediction model with disease progression or death due to OSCC among 69 OSCC patients in the MDACC dataset.

Variable	HR*	SE	p-value	95% CI	
Risk score	1.59	0.28	0.008	1.13	2.23
Age	0.98	0.01	0.294	0.96	1.01
Gender	0.93	0.49	0.894	0.33	2.60
Stage I/II vs. III/IV	1.65	0.97	0.388	0.53	5.19

* hazard ratio from a multivariable Cox regression model including risk score, age, gender, and tumor stage.

### Correlation between Affymetrix expression values and qRT-PCR

We found good correlations between Affymetrix expression values and qRT-PCR for the five genes we tested. The Pearson's correlations for *OCC1*, *DSE*, *ATCN1*, *RRAS2*, and *ITGA3* were 0.90, 0.73, 0.80, 0.67, and 0.87, respectively. The p-values for the correlations were <0.0001 for all five genes.

### Pathway analyses

Results of GSEA of the 71 probe sets show that the most prominent biologic pathway belongs to the integrin cell surface interactions pathway. The complete list of the pathways is presented in Table S2. When we compared the 71 probe sets to the 131 probe sets that we previously reported to be associated with survival of OSCC patients [55], we found eight genes (*KLF7*, *OSMR*, *PDPN*, *PADI1*, *CLEC3B*, *COL7A1*, *COL27A1*, and *NETO2*) that overlapped between the two gene lists. GSEA showed three pathways (Integrin cell surface interactions, ECM-receptor interaction, and focal adhesion) common to both gene lists.

## Discussion

OSCC has a high mortality rate, with a 5-year survival rate of 30–50% for late stage cancer (www.cancer.org). Fortunately, the 5-year survival rate exceeds 80% in early stage cancer. Thus, early detection or prevention of disease progression may help improve survival of OSCC patients. Identifying the key genes that play an important role in the progression of the carcinogenesis process may have potential clinical implications, e.g., as targets for OSCC prevention or treatment, or as biomarkers for early detection or prediction of disease progression. Our study is unique in that we collected not only normal oral mucosae from controls and tumor samples from OSCC, but we also collected uninvolved oral samples from OSCC patients. This design provided an opportunity to study the effect of field cancerization by comparing gene expression between normal oral mucosae from controls and uninvolved oral mucosae from OSCC patients, a comparison which may help identify genes that play a role in a very early stage of carcinogenesis. In addition, by comparing gene expression of uninvolved oral samples to that of tumor samples, we can further select genes that not only play a role at an early stage but that also play a role in the later stage of neoplastic development. We believe that the genes found through both of these comparisons are important in the progression of normal mucosa to cancer.

A limitation of this study is that the uninvolved oral samples from OSCC patients were not processed at the same time as normal oral samples and tumor samples: thus a batch effect is a potential issue to consider. We attempted to investigate and minimize the batch effect in several ways. First, we re-processed some tumor samples along with uninvolved oral samples and compared the gene expression to that of the previously processed tumor samples from the same patients. The results showed a good correlation of the gene expression between the re-processed samples and the samples previously processed. Second, we examined the quality of all arrays together to detect batch effects, and we then normalized all arrays together. Third, we performed a first step analysis to compare gene expression between tumor samples and normal oral samples from controls. The benefit from this first step analysis was that it minimized the penalty from multiple comparisons for the next step by reducing the number of genes to be further studied from more than 50,000 probe sets to approximately 2,500 “OSCC-related” probe sets. In addition, since tumor samples and normal oral samples were processed simultaneously with each batch containing both tumor sample and normal oral sample, the identification of these 2,596 probe sets from which the 20 final probe sets were determined were not affected by batch effect. It is possible that, by oversampling the uninvolved oral mucosae from patients who later developed local recurrence, we may have detected more differentially expressed genes between normal mucosa and uninvolved mucosa than had we selected the latter at random. However, a separate analysis to investigate whether the gene expression of uninvolved mucosa from patients with local recurrence differ from that of patients without local recurrence showed no significant difference in gene expression between the two groups (data not shown).

We identified 71 probe sets that showed dysregulation in the uninvolved oral samples and that showed even higher level of dysregulation in tumor samples. As mentioned earlier, one potential clinical implication of these genes is to predict disease progression. Thus we further tested whether some of the 71 probe sets were associated with disease progression or death due to OSCC and built a prediction model based on these genes. The results that 20 genes were associated with disease progression/OSCC-related death and can be used to predict disease progression independent of age, sex, tumor staging, and high-risk HPV status support our hypothesis.

We validated our results using data from 74 OSCC patients recruited at the MDACC. In the MDACC cohort, we found a significant association between disease progression/OSCC-related death and the risk score calculated from the prediction model based on the Cox model incorporating 20 probe sets. This association was independent of age, gender, and tumor stage. Moreover, the prediction model with risk score plus stage was better than the model with stage alone, suggesting that adding risk score to tumor stage improves the prediction of disease progression or OSCC-related death. To the best of our knowledge, this is the first gene signature using microarray data to predict disease progression/OSCC-related death that has been validated in an independent dataset from a different institution. One limitation in the MDACC is the lack of information on HPV status. Patients with HPV-positive tumors are more likely to have better survival, and HPV-positive tumors are more commonly found in the oropharynx [56], [57]. Among the 69 MDACC tumor samples, only three tumors were from the oropharynx. Thus, it is unlikely that HPV status would confound the association between risk scores and disease progression/OSCC-related death in the MDACC dataset. The fact that the tumor samples from the MDACC were frozen samples suggested that the use of this risk score is not limited to the RNAlater™-treated samples only.

Another potential use of the 20 or the 71 probe sets is to predict which premalignant lesions are likely to progress to cancer. Future study is needed to address this potential. In addition to prediction of disease progression, some of the 71 probe sets may serve as targets for detection or treatment of OSCC. For instance, SART2 (squamous cell carcinoma antigen recognized by T-cells 2) protein was found to be overexpressed in several types of cancer but not in normal cells [58]. One potential study would be to investigate the level of SART2 protein in saliva or oral rinse to determine whether it could help improve detection of OSCC, especially those that are located in areas that are difficult to visualize. Since SART2 is a tumor antigen recognized by cytotoxic T cells, it could potentially serve as a target for cancer immunotherapy as well. SART2-derived peptide has been shown to be immunogenic in hepatocellular carcinoma patients [59]. Further investigation is needed to explore the potential use of SART2 as a tumor marker or as a target for cancer immunotherapy for OSCC patients. Another gene that has been investigated as a potential anti-cancer target is Integrin α3β1 [60]. Genes in the Integrin family have functional roles in migration/invasion of tumor cells [61]–[63]. Among the 71 probe sets, four were genes in the Integrin family (*ITGA3*, *ITGAV*, *ITGB6*, and *ITGA6*). The most prominent pathway for the 71 probe sets is Integrin cell surface interaction pathway. Our results lend support to the important role of Integrins in cancer.

Previously, we reported that a 131 gene expression signature provided high discrimination between OSCC samples and normal oral mucosae from controls, and it was associated with OSCC-specific mortality [55]. Most of the 131 probe sets were in the list of 2,596 probe sets differentially expressed between tumor samples and normal oral samples in the current analyses. However, the difference in selection criteria provided different gene lists. The 131 probe sets were selected based on the most significant differences in gene expression between tumor and normal sample but the 71 probe sets were selected by emphasizing their potential involvement in both early and late stage of carcinogenesis.

In conclusion, we found dysregulation of gene expression of 71 probe sets, corresponding to 61 known genes, occurring early in uninvolved oral mucosae from OSCC patients, and the level of dysregulation was even higher in tumor samples. Dysregulation in the expression of 20 of the 71 probe sets was associated with disease free survival of OSCC patients. The result was validated in an independent dataset from the MDACC. If further confirmed in future studies, the expression of these genes has the potential to be developed into a clinical test. Such a test could help physicians to identify patients who need more aggressive treatment or frequent follow-up.

## Supporting Information

Table S1 **Seventy-one probe sets dysregulated in uninvolved oral samples and tumor samples of OSCC patients compared to normal oral mucosa from non-cancerous patients.**
(DOCX)

Table S2 **Gene set enrichment analysis identified pathways of 71 probe sets dysregulated in both uninvolved oral samples and cancer samples from OSCC patients.**
(DOCX)
