# Supplementary material for: Gene Expression in Uninvolved Oral Mucosa of OSCC Patients Facilitates Identification of Markers Predictive of OSCC Outcomes
Source: PLoS One. 2012 Sep 28;7(9):e46575. doi: 10.1371/journal.pone.0046575 (PMC3460916; doi:10.1371/journal.pone.0046575)
Supplement: Table S1 — Seventy-one probe sets dysregulated in uninvolved oral samples and tumor samples of OSCC patients compared to normal oral mucosa from non-cancerous patients. (DOCX) [file pone.0046575.s001.docx]

**Supplement Table 1**. Seventy-one probe sets dysregulated in uninvolved oral samples and tumor samples of OSCC patients compared to normal oral mucosa from non-cancerous patients, University of Washington Affiliated Medical Centers, 2003-2010

| Probe set ID | Gene Symbol | Gene Title | Fold difference (tumor vs. normal) |
| --- | --- | --- | --- |
| 231867_at | ODZ2 | odz, odd Oz/ten-m homolog 2 (Drosophila) | 7.2 |
| 221898_at | PDPN | podoplanin | 6.3 |
| 213110_s_at | COL4A5 | collagen, type IV, alpha 5 | 5.9 |
| 217820_s_at | ENAH | enabled homolog (Drosophila) | 5.1 |
| 225105_at | OCC1 | overexpressed in colon carcinoma-1 | 5.1 |
| 225288_at | COL27A1 | collagen, type XXVII, alpha 1 | 4.9 |
| 226535_at | ITGB6 | integrin, beta 6 | 4.7 |
| 218717_s_at | LEPREL1 | leprecan-like 1 | 4.4 |
| 226448_at | FAM89A | family with sequence similarity 89, member A | 4.4 |
| 201505_at | LAMB1 | laminin, beta 1 | 4.3 |
| 235683_at | SESN3 | sestrin 3 | 4.1 |
| 201250_s_at | SLC2A1 | solute carrier family 2 (facilitated glucose transporter), member 1 | 4.0 |
| 213139_at | SNAI2 | snail homolog 2 (Drosophila) | 4.0 |
| 211651_s_at | LAMB1 | laminin, beta 1 | 3.7 |
| 218888_s_at | NETO2 | neuropilin (NRP) and tolloid (TLL)-like 2 | 3.7 |
| 205122_at | TMEFF1 | transmembrane protein with EGF-like and two follistatin-like domains 1 | 3.7 |
| 222774_s_at | NETO2 | neuropilin (NRP) and tolloid (TLL)-like 2 | 3.6 |
| 1554018_at | GPNMB | glycoprotein (transmembrane) nmb | 3.5 |
| 217312_s_at | COL7A1 | collagen, type VII, alpha 1 | 3.5 |
| 201474_s_at | ITGA3 | integrin, alpha 3 (antigen CD49C, alpha 3 subunit of VLA-3 receptor) | 3.4 |
| 201656_at | ITGA6 | integrin, alpha 6 | 3.4 |
| 225258_at | FBLIM1 | filamin binding LIM protein 1 | 3.3 |
| 218847_at | IGF2BP2 | insulin-like growth factor 2 mRNA binding protein 2 | 3.3 |
| 206581_at | BNC1 | basonuclin 1 | 3.2 |
| 1552277_a_at | MSANTD3 | Myb/SANT-like DNA-binding domain containing 3 | 3.2 |
| 204136_at | COL7A1 | collagen, type VII, alpha 1 | 3.1 |
| 202351_at | ITGAV | integrin, alpha V (vitronectin receptor, alpha polypeptide, antigen CD51) | 3.1 |
| 201976_s_at | MYO10 | myosin X | 3.1 |
| 221538_s_at | PLXNA1 | plexin A1 | 2.9 |

**Supplement Table 1** continued

| Probe set ID | Gene Symbol | Gene Title | Fold difference (tumor vs. normal) |
| --- | --- | --- | --- |
| 209935_at | ATP2C1 | ATPase, Ca++ transporting, type 2C, member 1 | 2.9 |
| 205796_at | TCP11L1 | t-complex 11 (mouse)-like 1 | 2.9 |
| 208636_at | ACTN1 | actinin, alpha 1 | 2.9 |
| 1558152_at | LOC100131262 | hypothetical LOC100131262 | 2.8 |
| 202599_s_at | NRIP1 | nuclear receptor interacting protein 1 | 2.8 |
| 204334_at | KLF7 | Kruppel-like factor 7 (ubiquitous) | 2.8 |
| 201249_at | SLC2A1 | solute carrier family 2 (facilitated glucose transporter), member 1 | 2.7 |
| 235492_at | RNF217 | ring finger protein 217 | 2.5 |
| 225150_s_at | RTKN | rhotekin | 2.5 |
| 212285_s_at | AGRN | agrin | 2.4 |
| 202872_at | ATP6V1C1 | ATPase, H+ transporting, lysosomal 42kDa, V1 subunit C1 | 2.3 |
| 1554008_at | OSMR | oncostatin M receptor | 2.3 |
| 204068_at | STK3 | serine/threonine kinase 3 | 2.3 |
| 218854_at | SART2 | squamous cell carcinoma antigen recognized by T cells 2 | 2.3 |
| 202066_at | PPFIA1 | protein tyrosine phosphatase, receptor type, f polypeptide (PTPRF), interacting protein (liprin), alpha 1 | 2.2 |
| 209011_at | TRIO | triple functional domain (PTPRF interacting) | 2.2 |
| 238933_at | IRS1 | insulin receptor substrate 1 | 2.2 |
| 203935_at | ACVR1 | activin A receptor, type I | 2.2 |
| 1554795_a_at | FBLIM1 | filamin binding LIM protein 1 | 2.2 |
| 1554016_a_at | C16orf57 | chromosome 16 open reading frame 57 | 2.2 |
| 209934_s_at | ATP2C1 | ATPase, Ca++ transporting, type 2C, member 1 | 2.2 |
| 202027_at | TMEM184B | transmembrane protein 184B | 2.2 |
| 214853_s_at | SHC1 | SHC (Src homology 2 domain containing) transforming protein 1 | 2.2 |
| 217788_s_at | GALNT2 | UDP-N-acetyl-alpha-D-galactosamine:polypeptide N-acetylgalactosaminyltransferase 2 (GalNAc-T2) | 2.2 |
| 212589_at | RRAS2 | related RAS viral (r-ras) oncogene homolog 2 | 2.1 |
| 224747_at | UBE2Q2 | ubiquitin-conjugating enzyme E2Q family member 2 | 2.0 |
| 228914_at | MSANTD3-TMEFF1 | MSANTD3-TMEFF1 readthrough | 2.0 |
| 202896_s_at | SIRPA | signal-regulatory protein alpha | 2.0 |
| 224791_at | ASAP1 | ArfGAP with SH3 domain, ankyrin repeat and PH domain 1 | 2.0 |
| 209081_s_at | COL18A1 | collagen, type XVIII, alpha 1 | 2.0 |

**Supplement Table 1** continued

| Probe set ID | Gene Symbol | Gene Title | Fold difference (tumor vs. normal) |
| --- | --- | --- | --- |
| 206335_at | GALNS | galactosamine (N-acetyl)-6-sulfate sulfatase | 2.0 |
| 225795_at | C22orf32 | chromosome 22 open reading frame 32 | -2.1 |
| 222368_at | no gene symbol | EST384442 MAGE resequences, MAGL Homosapiens cDNA | -2.2 |
| 215913_s_at | GULP1 | GULP, engulfment adaptor PTB domain containing 1 | -2.2 |
| 234233_s_at | no gene symbol | cDNA FLJ20924 fis, clone ADSE00928 | -2.5 |
| 220962_s_at | PADI1 | peptidyl arginine deiminase, type I | -2.7 |
| 207057_at | SLC16A7 | solute carrier family 16, member 7 (monocarboxylic acid transporter 2) | -3.1 |
| 206453_s_at | NDRG2 | NDRG family member 2 | -3.8 |
| 228335_at | CLDN11 | claudin 11 | -5.5 |
| 1569608_x_at | no gene symbol | clone IMAGE: 4720764 | -5.8 |
| 210085_s_at | ANXA9 | annexin A9 | -7.1 |
| 205200_at | CLEC3B | C-type lectin domain family 3, member B | -7.3 |
