# Supplementary material for: Gene Expression in Uninvolved Oral Mucosa of OSCC Patients Facilitates Identification of Markers Predictive of OSCC Outcomes
Source: PLoS One. 2012 Sep 28;7(9):e46575. doi: 10.1371/journal.pone.0046575 (PMC3460916; doi:10.1371/journal.pone.0046575)
Supplement: Table S2 — Gene set enrichment analysis identified pathways of 71 probe sets dysregulated in both uninvolved oral samples and cancer samples from OSCC patients. (DOCX) [file pone.0046575.s002.docx]

**Supplement Table 2.** Gene set enrichment analysis identified pathways of 71 probe sets deregulated in both uninvolved oral samples and cancer samples from OSCC patients, University of Washington Affiliated Medical Centers, 2003-2010.

| **Description of gene sets** | **K*** | **k**** | **p value^** |
| --- | --- | --- | --- |
| Genes involved in Integrin cell surface interactions | 81 | 5 | 6.39E-04 |
| Genes involved in Cell surface interactions at the vascular wall | 94 | 5 | 1.26E-03 |
| Vitamin C in the Brain | 11 | 2 | 3.87E-03 |
| Arrhythmogenic right ventricular cardiomyopathy (ARVC) | 76 | 4 | 4.11E-03 |
| Integrin Signaling Pathway | 78 | 4 | 4.51E-03 |
| ECM-receptor interaction | 84 | 4 | 5.88E-03 |
| Small cell lung cancer | 84 | 4 | 5.88E-03 |
| Genes involved in Cell junction organization | 84 | 4 | 5.88E-03 |
| Focal adhesion | 201 | 6 | 7.56E-03 |
| Genes involved in Cell-extracellular matrix interactions | 16 | 2 | 8.20E-03 |
| Genes involved in Basigin interactions | 25 | 2 | 1.95E-02 |
| Genes involved in Hemostasis | 274 | 6 | 3.05E-02 |
| Hypertrophic cardiomyopathy (HCM) | 85 | 3 | 3.71E-02 |
| Regulation of actin cytoskeleton | 216 | 5 | 3.85E-02 |
| Integrin Signaling Pathway | 38 | 2 | 4.26E-02 |
| Dilated cardiomyopathy | 92 | 3 | 4.52E-02 |

* A total number of genes in each gene set

** number of genes overlap with genes in 71 gene list.

^ p-value calculated based on the hypergeometirc distribution (identical to the corresponding one-tailed version of Fisher’s exact test).
